# Superconducting and charge-ordered states in the anisotropic *t*–*J*–*U* model

**DOI:** 10.1038/s41598-024-51829-7

**Published:** 2024-01-16

**Authors:** Yifan Feng, Jie Lou, Yan Chen

**Affiliations:** Department of Physics and State Key Laboratory of Surface Physics, Fudan University, Shanghai, 200433 China

**Keywords:** Condensed-matter physics, Superconducting properties and materials, Theory and computation, Computational methods

## Abstract

Motivated by the effect of symmetry breaking in cuprates superconductors YBa$_2$Cu$_3$O$_{7-\delta }$, we employ the renormalized mean-field theory to study the presence of uniform superconducting and charge-ordered states in two anisotropic *t*–*J*–*U* models, either with hopping strength anisotropy or antiferromagnetic interaction anisotropy. In the case of uniform superconducting state, compared with the isotropic *t*–*J*–*U* model with only $d_{x^2-y^2}$-wave superconducting state, there is an additional *s*-wave superconducting state in the model with hopping strength anisotropy. Meanwhile, the hopping anisotropy may enhance the critical Coulomb interaction $U_c$ at the Mott insulator to the Gossamer superconductor transition point, and strong hopping anisotropy may weaken the superconducting state. In the case of a charge-ordered state, hopping anisotropy may suppress the amplitude of the charge density waves and pair density waves, which originate from local Coulomb interactions. These results indicate that the effects of hopping anisotropy and local Coulomb interactions are competitive. Moreover, the antiferromagnetic interaction anisotropy only weakly suppresses the superconducting gap and density wave amplitude. Our results show that the *t*–*J*–*U* model with hopping anisotropy is qualitatively consistent with experimental superconducting pair symmetry and charge density waves in the YBa$_2$Cu$_3$O$_{7-\delta }$ system.

## Introduction

Due to the strong correlations between charge and spin degrees of freedom, high-temperature cuprate superconductors exhibit exotic physical properties^1,2^. The electronic and magnetic properties of these materials have been studied extensively, with the primary role played by the common CuO$_2$ plane. Among these cuprate superconductors, YBa$_2$Cu$_3$O$_{7-\delta }$(YBCO) material has a one-dimensional CuO chain to the prevalent two-dimensional CuO$_2$ plane. Meanwhile, many experiments revealed the physical properties with anisotropy of YBCO via Nernst^3,4^, transport^5^ and inelastic-neutron-scattering measurements^6^. Therefore, its pairing symmetry and charge order differ from materials without a CuO chain. Angle-resolved photoelectron spectroscopy^7,8^, inelastic light scattering^9^ and phase-sensitive measurement^10,11^ show that the superconducting energy gap will vary in different directions due to lattice anisotropy, giving $(d_{x^2-y^2}+s)$-wave superconducting pairing. Another class of states of interest in the underdoped to the optimal doping region is called charge order states. X-ray scattering^12–14^, X-ray diffraction^15–17^, and sound velocity measurements^18^ experiments support the presence of an incommensurate charge density wave (CDW) state with a wave vector around 0.31 in YBCO, which is different from the commensurate charge density wave with a wave vector of 0.25 in lanthanum-based “214”-type cuprates^19–23^.

The CuO$_2$ plane plays an essential role in the formation of superconductivity in cuprates, and its fundamental features can be described by the *t*–*J* model^24,25^. The projection operator in the *t*–*J* model excludes double occupancy at one site, reflecting a strong correlation effect. In particular, Gutzwiller approximation in renormalized mean-field theory (RMFT)^26^ uses statistical weight factors to handle the projection operator, and $d_{x^2-y^2}$-wave superconducting state and some charge-ordered states have been found^27–31^. As an effective model in the strong *U* limit of the Hubbard model, the *t*–*J* model serves as a minimal model to describe the high-temperature superconductivity. However, in cuprates, the ratio of Coulomb interaction to bandwidth deviates from the strong correlation limit, and the possibility of double occupancy cannot be excluded entirely. Therefore, it is necessary to consider the *t*–*J*–*U* model^32,33^, which contains both the kinetic energy term, superexchange term, and the on-site Coulomb repulsion term. It can describe a weak and intermediate interacting strength system. When $U=0$, it corresponds to noninteracting tight-binding model, and in the limit $U\rightarrow \infty$, it is reduced to the *t*–*J* model. Several methods are used to solve this model, such as variational Monte Carlo^34^, RMFT^34–36^, density matrix renormalization group^32,37^ and diagrammatic expansion Gutzwiller wave-function method^38,39^. Compared with the experimental data, the *t*–*J*–*U* model is more suitable than the *t*–*J* model to describe cuprates^38^.

Several models have been proposed to describe the electronic states in YBCO materials, such as the multiband model containing CuO$_2$ planes with CuO chains^40–44^. Considering the orbital hybridization between the plane and the chain, the multiband model can be reduced to an anisotropic *t*–*J* model in the CuO$_2$ plane^45–50^. To describe the anisotropy in YBCO materials more precisely, we consider two anisotropic *t*–*J*–*U* models in this paper and use the RMFT method to study the superconducting pair symmetry and charge-ordered states. We find $(d_{x^2-y^2}+s)$-wave superconducting state consistent with the experiment and two CDW states accompanied by pair density wave (PDW). One is the antiphase charge density wave (AP-CDW) state with alternating positive and negative pair field distribution. Moreover, the other is the nodal pair density wave (nPDW) state with a non-zero net pairing field. Both density wave states are suppressed with the enhancement of the hopping anisotropy. We also calculate the local density of states (LDOS) in the nPDW states, obtain the strength of superconducting coherence *D*, and find that it oscillates synchronously with the hole density in real space.

## Results

### Hopping anisotropy

#### Phase diagram of superconducting state

**Figure 1:**
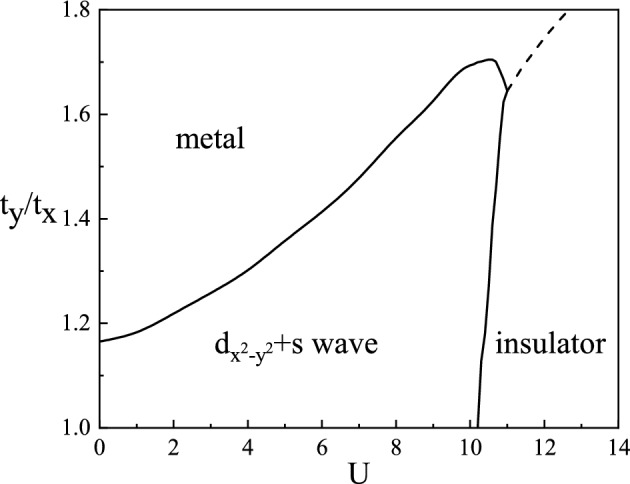
Phase diagram at half-filling.

As mentioned above, we use anisotropic *t*–*J*–*U* models to study superconducting and charge-ordered states in YBCO materials. RMFT has been applied to analyze strongly correlated electrons. We introduce four variational order parameters, electron density $n_i$, doublon density $d_i$, pairing field $\Delta _{i j \sigma }^{\nu }$, and bond field $\chi _{i j \sigma }^{\nu }$. Details are discussed in “Methods” section. First, we focus on the uniform state in the anisotropic hopping model at the half-filled case. Figure 1 shows the phase diagram in the parameter space *U* and $t_y/t_x$. The horizontal axis is Coulomb repulsion, reflecting the strength of the correlation, and the vertical axis is the ratio of the hopping in the *y* direction to the hopping in the *x* direction, reflecting the hopping anisotropy. When $t_y/t_x=1$, it is an isotropic *t*–*J*–*U* model, and *d*-wave pair state appears at $U<10.23$. In real space, the superconducting gap in the *x* and *y* directions are equal in size and opposite in sign.

When the anisotropy of the hopping strength is small, and the Coulomb repulsion weakens, an admixed $d_{x^2-y^2}$+*s* pair state will appear in the system. This is because the anisotropy hopping makes the superconducting gap unequal in the *x* and *y* direction, which is consistent with the existence of ($d_{x^2-y^2}$+*s*)-wave superconductivity in anisotropy YBa$_2$Cu$_3$O$_{7-\delta }$. Gradually increasing the anisotropy of the hopping strength, the superconducting state is suppressed. Still, there is a finite effective carrier density *d*, and finally, a second-order phase transition occurs and transforms into a metallic state. When the hopping anisotropy is small and the Coulomb repulsion gradually increases, the first-order phase transition between the superconductor and insulator occurs, and the system enters the strong correlation region from the weak correlation region. It is noted that anisotropy will delay the system from the superconducting phase to the insulating phase, that is, the increase of $U_c$. To a certain extent, there is a competitive effect between the influence of hopping anisotropy and Coulomb repulsion on uniform state.

**Figure 2:**
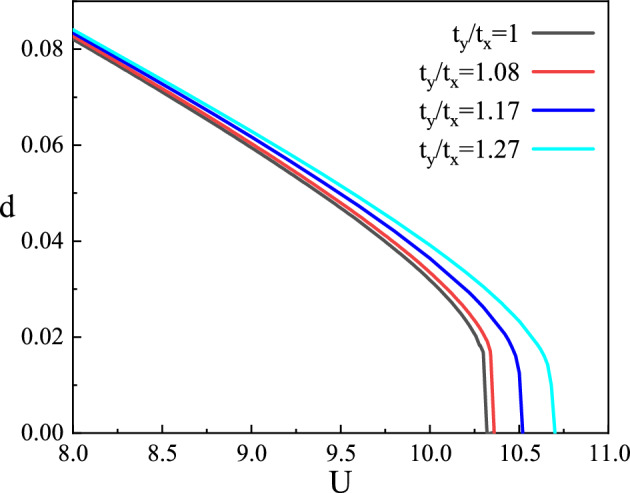
The doublon density *d* as a function of *U* at half-filling.

In Fig. 2, we show the doublon density for different hopping strength anisotropy as a function of Coulomb repulsion at half-filling. When $t_y/t_x=1$, the doublon density decreases continuously with the increase of the Coulomb repulsion until $U_c = 10.23$ and tends to a finite value $d_c = 0.02$. Then the doublon density jumps to zero, and the model is equivalent to the *t*–*J* model, indicating a first-order Mott transition at this time. When the Coulomb repulsion weakens, the variation trends of doublon density with Coulomb repulsion are the same under different anisotropy. When the Coulomb repulsion reaches the vicinity of $U_c$, as the hopping strength anisotropy increases, $U_c$ also increases, and the existing area of the Gossamer superconducting state become wider. Meanwhile, the doublon density discontinuity becomes smaller and tends to change continuously to zero, weakening the first-order phase transition characteristics. It is noted that when $t_y/t_x = 1.27$, there is a phase transition from metallic phase to superconducting phase and finally to Mott insulating phase in the system with the increase of *U*. When the phase transition from metallic state to superconducting state occurs, the variation trend of doublon density does not change, which is a second-order phase transition.

**Figure 3:**
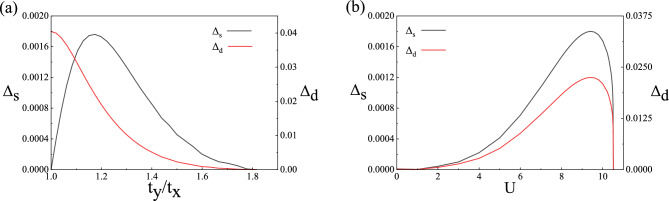
Superconducting order parameter (**a**) superconducting order parameter with $d_{x^2-y^2}$-wave $\Delta _d$ and *s*-wave $\Delta _s$ as a function of anisotropy $t_y/t_x$ for $U=9$. (**b**) superconducting order parameter with $d_{x^2-y^2}$-wave $\Delta _d$ and *s*-wave $\Delta _s$ as a function of Coulomb repulsion *U* for $t_y/t_x=1.17$.

In Fig. 3a, we show $d_{x^2-y^2}$-wave superconducting gap $\Delta _d$ and *s*-wave superconducting gap $\Delta _s$ as a function of anisotropy parameter $t_y/t_x$ for $U=9$. With the increase of $t_y/t_x$, $\Delta _d$ decreases, but $\Delta _s$ gradually increases from zero to a maximum value and then decreases again. $\Delta _s$ reaches its maximum at $t_y/t_x=1.17$ and $\Delta _s/\Delta _d=0.09$. The ratio of *s*-wave to $d_{x^2-y^2}$-wave gap is consistent with the phase-sensitive experimental results^10^. When $t_y/t_x>1.8$, $\Delta _d$ and $\Delta _s$ decrease smoothly to zero. However, the superconducting gap in the *x* and *y* directions always decreases with increasing hopping anisotropy. Excessive hopping anisotropy may lead to the gap closing prematurely in one direction. Figure 3b shows $\Delta _d$ and $\Delta _s$ as a function of Coulomb repulsion *U* for $t_y/t_x=1.17$. As *U* increases, $\Delta _s$ and $\Delta _d$ reach a maximum value at $U=9.4$ and $\Delta _s/\Delta _d=0.09$ discontinuously decrease to zero at $U_c=10.52$, with the same trend as the order parameter in the isotropic *t*–*J*–*U* model. There is a first-order phase transition from the Gossamer superconductor to the Mott insulator at $U_c$. It can be seen that the anisotropy hopping *t*–*J*–*U* model can well describe superconducting pairing symmetry and gap size of YBCO materials.

#### Charge-ordered state

Next, we focus on the charge-ordered state. In the *t*–*J*–*U* model, charge-ordered states appear at $U>9$, commensurate and incommensurate charge-ordered states originating from local Coulomb interactions are strongly suppressed at the region where anisotropy appears. Unlike the ($d_{x^2-y^2}$+*s*)-wave pair in the uniform state in the hopping anisotropy *t*–*J*–*U* model, there is only the *d*-wave gap with spatial modulation, and the *s*-wave gap is almost zero.

**Figure 4:**
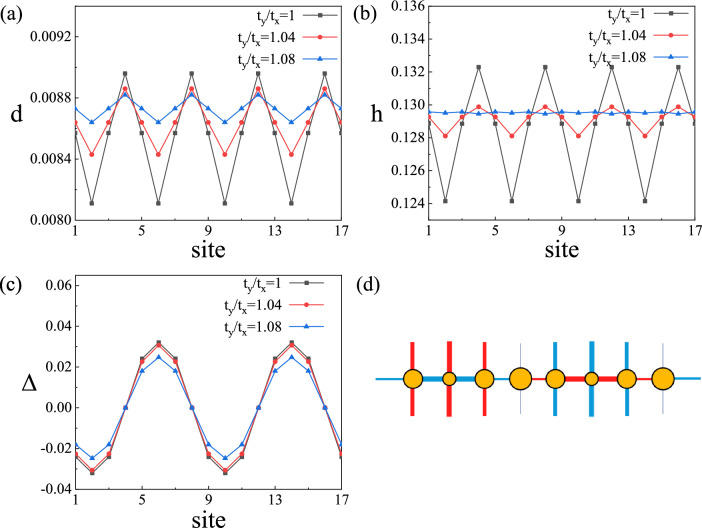
Features of the AP-CDW state at $U = 15$ and $\delta =0.12$. The spatial variations of (**a**) doublon density, (**b**) hole density, and (**c**) superconducting order parameter in the system with lattice sites 1–17 in *x* direction. (**d**) Schematic illustration of modulations for the AP-CDW state. The diameter of the orange circles indicates the local hole density. The width of a bond around each site indicates the local pairing field and sign is positive (negative) for red (blue).

When $t_y/t_x$ anisotropy is considered, in the strongly correlated region $U>9$, there are also the AP-CDW state and nPDW state similar to that in the isotropic *t*–*J*–*U* model. There are two effective carrier doublon $d_i$ and hole $h_i$ in the model, and the relationship with the doping density $\delta _i$ is $h_i=1-n_i+d_i=\delta _i+d_i$. In the strong correlation region $U=15$, we first present the AP-CDW state results at doping density $\delta =0.12$ in Fig. 4.The modulations are unidirectional stripe pattern which is along *y* direction. Figure 4d shows a schematic illustration of modulations for AP-CDW. Figure 4a shows the variation of doublon density $d_i$ at lattice sites $i=1$ to $i=17$, and Fig. 4b shows the variation of hole density $h_i$ at the same sites. The modulation of $d_i$ and $h_i$ have the same period of 4, and they change synchronously. Figure 4c shows the variation of gap order parameter $\Delta _i$ at the same sites, and it has a period of 8 twice that of $d_i$ and $h_i$. The gap order parameter changes sign once every half period. Moreover, the system has domain walls where the gap order parameter vanishes, and doublon and hole reach a maximum. In this case, the net pairing is 0. The very similar behavior of $d_i$ and $h_i$ originates from the strong correlation effect in the system, indicating the duality of doublon and hole. With the enhancement of hopping anisotropy, the oscillations of both charge and pair density wave states are significantly suppressed, and the suppression of hole and doublon fluctuations is more substantial. Still, the modulation period is not changed, and eventually, the system enters a uniform state. Density wave states originate from stronger Coulomb interactions, while hopping anisotropy can destroy density wave states, indicating that the effects of hopping anisotropy and correlation compete with each other.

**Figure 5:**
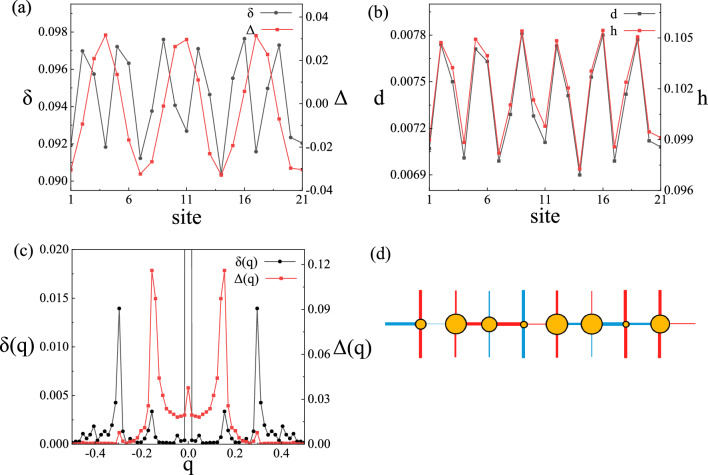
Features of the nPDW state at $t_y/t_x=1.08$, $U = 15$ and $\delta =0.094$. The spatial variations of (**a**) a variation of doping density ($\delta$, left axis) and superconducting order parameter ($\Delta$, right axis) with lattice sites 1–21 in *x* direction. (**b**) A variation of doublon density (*d*, left axis) and hole density (*h*, right axis) with lattice sites 1–21 in *x* direction. (**c**) Fourier transform of hole density and superconducting order parameter. (**d**) Schematic illustration of modulations for the nPDW state. The diameter of the orange circles indicates the local hole density. The width of a bond around each site indicates the local pairing field and sign is positive (negative) for red (blue).

When the hopping strength anisotropy is introduced, the nPDW state also exists in the system, as shown in Fig. 5. In this case, the spatial modulation of the charge density wave and the pair density wave is incommensurate. Figure 5d shows a schematic illustration of modulations for nPDW. Figure 5a,b show the local density $\delta _i$, $\Delta _i$, $d_i$ and $h_i$ at lattice sites $i=1$ to $i=21$. The system has a more complex density wave modulation. $\delta _i$, $d_i$ and $h_i$ are modulated synchronously. They reach the maximum and minimum values at the same time. And $\Delta _i$ drops near zero where $\delta _i$, $d_i$ and $h_i$ reach the maximum. $\delta (q)$ and $\Delta (q)$ in Fig. 5c are the Fourier transform of $\delta$ and $\Delta$. Furthermore, the spatial average value of the superconducting order parameter is not zero, that is, $\Delta (q=0)\ne 0$ after Fourier transform. The superconducting order parameter $\Delta (q)$ peaks at $q=0.15$, and the doping density $\delta (q)$ peaks at $q=0.3$, which is close to the experimentally observed YBCO charge density wave vector of 0.31^15^. Similarly, when the hopping anisotropy is too strong, the nPDW state is destroyed, and the system returns to the uniform state. With the increase of anisotropy $t_y/t_x$, the pairing field in the *y* direction decreases, leading to a transition from density wave state in the *x* direction to the uniform state.

**Figure 6:**
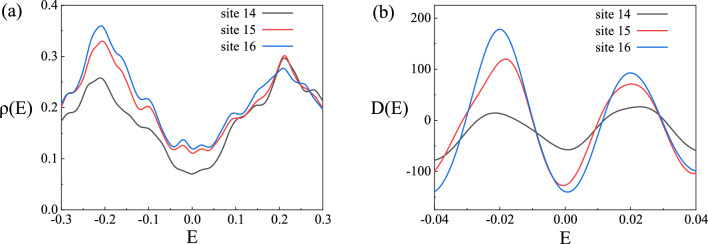
Features of the nPDW state at $t_y/t_x=1.08$, $U = 15$ and $\delta =0.094$. (**a**) Local density of states $\rho (E)$ as a function of *E* at sites 14–16. (**b**) The strength of superconducting coherence *D*(*E*) as a function of *E* at sites 14–16 in the system.The amplitude of $\Delta _i$ reaches maximum at site 14 and close to zero at site 16.

We also give the local density of states $\rho (E)$ and superconducting coherence strength *D*(*E*) of nPDW states at lattice sites $i=14$ to $i=16$ after introducing hopping strength anisotropy, as shown in Fig. 6. At site $i=14$, $\delta _i$, $d_i$ and $h_i$ reach the minimum, and the amplitude of $\Delta _i$ reach the maximum. At site $i=16$, $\delta _i$, $d_i$ and $h_i$ reach the maximum, and the amplitude of $\Delta _i$ is close to zero. Due to the $d_{x^2-y^2}$-wave pairing symmetry and the existence of the non-zero component of the superconducting order parameter at $q=0$, the LDOS exhibits a V-type structure near the Fermi energy and remains non-zero at $E=0$. It first decreases, then increases, and then decreases with the change of the lattice position, which has the same modulation as the hole density. The strength of superconducting coherence can be characterized by the negative of the second derivative of the LDOS $D(E)=-\rho ''(E)$^36^. The height of the peak reflects the sharpness of the superconducting coherence peak, and it has the same modulation with $\delta _i$, $d_i$, and $h_i$. From sites 14 to 16, the peak height and $\delta _i$ increase simultaneously.

#### Antiferromagnetic interaction anisotropy

**Figure 7:**
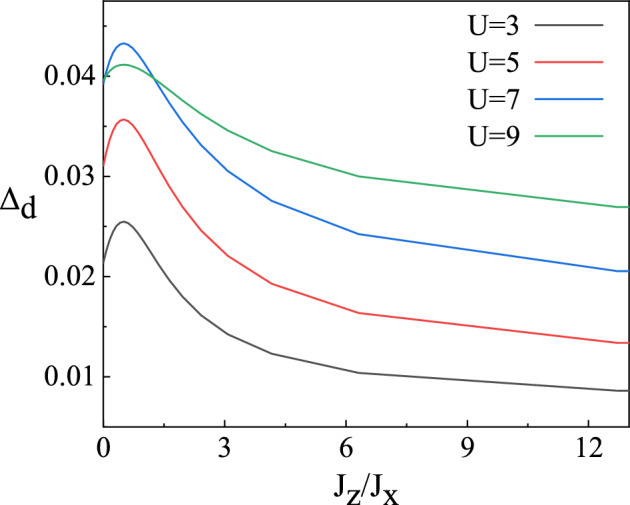
*d*-wave order parameter $\Delta _d$ at half-filling for several values of U.

In addition to the novel physics due to the hopping anisotropy in real space, we further consider intrinsic anisotropy in spin space as a theoretical conceptual extension of the *t*–*J*–*U* model, which breaks SU(2) symmetry. In this section, we consider the anisotropic antiferromagnetic interaction model. Now we study the uniform states in the *t*–*J*–*U* model with antiferromagnetic interaction anisotropy. Compared with the results of the isotropic *t*–*J*–*U* model, the pairing symmetry and critical correlation strength $U_c$ of the superconducting phase are virtually not changed. There is still a pure $d_{x^2-y^2}$-wave gap in the system, and the magnitude of antiferromagnetic interaction anisotropy only changes the gap size. The variation of $d_{x^2-y^2}$-wave superconducting gap with the anisotropy of the antiferromagnetic interaction is shown in Fig. 7. The width of the superconducting gap varies with $J_z/J_x$ in a small range, and superconducting gap reaches its maximum as the anisotropy disappears. In the *t*–*J*–*U* model, the influence of antiferromagnetic interaction anisotropy on the superconducting state is far less than that of hopping anisotropy and Coulomb repulsion strength. So the antiferromagnetic interaction anisotropy does not play a dominant role in uniform states. For the superconducting state in YBCO materials, the *t*–*J*–*U* model with antiferromagnetic interaction anisotropy does not represent the basic features.

**Figure 8:**
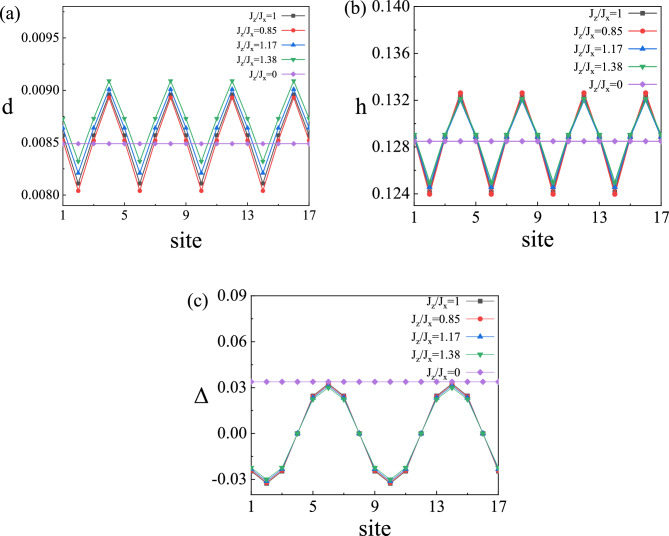
Features of the AP-CDW state at $U = 15$ and $\delta =0.12$ with lattice sites 1–17 in *x* direction. (**a**) Variation of doublon density in the system. (**b**) Variation of hole density in the system. (**c**) Variation of the superconducting order parameter in the system.

Next, we consider the effect of antiferromagnetic interaction anisotropy on charge-ordered states. The parameters of the pair density wave and the charge density wave states are shown in Fig. 8. Figure 8a shows the variation of doublon density $d_i$, Fig. 8b shows the variation of hole density $h_i$, and Fig. 8c shows the variation of gap order parameter $\Delta _i$. There are charge-ordered states similar to the *t*–*J*–*U* model with hopping anisotropy, pair density wave state with a period of 8 and charge density wave state with a period of 4, and pairing symmetry is $d_{x^2-y^2}$-wave. Schematic illustration of modulation is similar to Fig. 4d. Hole and doublon are also modulated synchronously, and the density wave state is suppressed when antiferromagnetic interaction anisotropy is introduced. Due to symmetry, there is a similar modulation amplitude for pair density waves state when $J_z/J_x=J_x/J_z=0.85$, while charge density waves state are symmetric concerning $J_z/J_x=1$. In the limit case $J_z/J_x=0$, density wave states are significantly suppressed, and only the uniform state exists. The amplitude of density wave states changes slowly with $J_z/J_x$, and the influence of antiferromagnetic interaction anisotropy on density wave states is weaker than that of hopping anisotropy. Moreover, the influence of antiferromagnetic interaction anisotropy on density wave states is weaker than that of hopping anisotropy. There is no density wave state with wave vector $q=0.3$.

## Conclusion

To describe the lattice anisotropy induced by CuO chains in YBCO materials and magnetic anisotropy, we study the *t*–*J*–*U* model with hopping strength anisotropy and antiferromagnetic interaction anisotropy in the framework of the renormalized mean-field theory. For the hopping strength anisotropy model, the superconducting pairing symmetry is still dominated by the $d_{x^2-y^2}$-wave, but with the deviation of the isotropic *t*–*J*–*U* model, the system will gradually have the pairing symmetry of the *s*-wave, and the ratio of *s*-wave to $d_{x^2-y^2}$-wave gap is $\Delta _s/\Delta _d=0.09$. The *t*–*J*–*U* model with hopping strength anisotropy can explain the pairing symmetry of the YBCO materials, compared with the experimental results^10^. Although $(d_{x^2-y^2}+s)$-wave superconducting state is induced by the presence of anisotropic hopping, the superconducting gap decreases with the increase of hopping anisotropy. The study of the three-band model through the diagrammatic expansion of the Gutzwiller wave function method also confirmed that there is a mixed *d*- and *s*-wave pairing when the four-fold rotational symmetry is broken^51^. In the case of large *U*, there are charge-ordered states in the *t*–*J*–*U* model with hopping anisotropy, which are AP-CDW and nPDW states. The nPDW state wave vector is in good agreement with the experiment^15^. Moreover, the hopping anisotropy significantly inhibits the amplitude of charge-ordered states. In this case, the suppression mechanism of hopping anisotropy on the charge-ordered states is similar to that of the superconducting state, which inhibits the amplitude of the density waves by weakening the pairing field in *y* direction. Recent experiments have found that applying uniaxial stress to cuprates distorts the structure and reduces CDW order^52,53^, similar to our calculations. This shows that uniaxial stress can also tune the hopping anisotropy $t_y/t_x$.

Further taking the intrinsic magnetic anisotropy into account, the *t*–*J*–*U* model with antiferromagnetic interaction anisotropy shows no new results. The effect of antiferromagnetic interaction anisotropy on the superconducting state is not very strong in the case of small *U* but makes the superconducting gap fluctuate in a small range. In the two anisotropic models, it is similar that the anisotropy will limit the size of the pairing field, and then affect the formation of the gap. In the large *U* area, antiferromagnetic interaction anisotropy has little effect on the amplitude of charge-ordered states. All charge-ordered states are suppressed in the limit case, leaving only uniform states. Both types of anisotropy will suppress the charge fluctuations. Therefore, the *t*–*J*–*U* model with hopping anisotropy can represent the essential physics of YBCO materials.

## Methods

We study two anisotropic *t*–*J*–*U* models, hopping anisotropy and antiferromagnetic interaction anisotropy. The *t*–*J*–*U* model with hopping anisotropy on a square lattice is given by

1 $$\begin{aligned} H=-\sum _{i\sigma }\left( t_x c_{i+x\sigma }^\dagger c_{i\sigma }+t_y c_{i+y\sigma }^\dagger c_{i\sigma }+ \text{ H.c. } \right) +J \sum _{\langle i j\rangle } {\varvec{S_{i}}} \cdot {\varvec{S_{j}}} +U\sum _{i}n_{i\uparrow }n_{i\downarrow }. \end{aligned}$$

where $t_x$ and $t_y$ are the hopping matrix element between the nearest neighbor in the *x* and *y* direction. The average of nearest-neighbor hopping $(t_x+t_y)/2$, as our energy unit, is set to 1. *J* term is the Heisenberg interaction between the nearest neighbor and is set to $J=1/3$. $c_{i\sigma }$ is electron annihilation operator with spin $\sigma =\pm$ at site *i* and ${\varvec{S_i}}$ is the spin-1/2 operator. $U>0$ is on-site Coulomb repulsion.

The *t*–*J*–*U* model with antiferromagnetic interaction anisotropy on a square lattice is given by

2 $$\begin{aligned} H=-\sum _{\langle ij\rangle , \sigma } t\left( c_{i \sigma }^{\dagger } c_{j \sigma }+ \text{ H.c. } \right) +\sum _{\langle ij\rangle } \left( \frac{J_x}{2}S_i^+ S_j^- + \frac{J_x}{2}S_i^- S_j^+ + J_zS_i^z S_j^z\right) +U\sum _{i}n_{i\uparrow }n_{i\downarrow }. \end{aligned}$$

where *t* term denotes the nearest-neighbor hopping matrix element, and it is set to be the energy unit. The nearest-neighbor antiferromagnetic Heisenberg interaction $J_x(J_z)$ satisfy $J_x^2+J_z^2=(1/3)^2$.

We take a partially projected variational trial wave function proposed by Laughlin^54^

3 $$\begin{aligned} \left| \Psi _{G S}\right\rangle= & {} \prod _{\alpha }\left| \Psi _{0}\right\rangle , \end{aligned}$$

4 $$\begin{aligned} \prod _{\alpha }= & {} \prod _{i}\left( 1-\alpha n_{i \uparrow } n_{i \downarrow }\right) . \end{aligned}$$

where $|\Psi _0\rangle$ is BCS superconducting state, and $\prod _{\alpha }$ is the projection operator, which partially projects out double occupancy on each site. $|\Psi _{GS}\rangle$ smoothly connects the BCS state with the RVB state. $\alpha = 0$ represents the BCS state and $\alpha = 1$ corresponds to the RVB state.

The projection operator incorporates strong electron correlations, which can be handled by the Gutzwiller approximation method, which considers the effect of projection through a set of statistical weight factors. Then the hopping term and the Heisenberg interaction in the partially projected state and unprojected BCS state are related by Gutzwiller renormalization factors

5 $$\begin{aligned}{} & {} \left\langle c_{i \sigma }^{\dagger } c_{j \sigma }\right\rangle =g_{i j}^{t}\left\langle c_{i \sigma }^{\dagger } c_{j \sigma } \right\rangle _{0}, \nonumber \\{} & {} \left\langle {\varvec{S_{i}}} \cdot {\varvec{S_{j}}}\right\rangle =g_{i j}^{s}\left\langle {\varvec{S_{i}}} \cdot {\varvec{S_{j}}}\right\rangle _{0}. \end{aligned}$$

The Gutzwiller renormalization factors on each site are given as

6 $$\begin{aligned} g_{i j}^{t}= & {} g_{i}^{t} g_{j}^{t}, \nonumber \\ g_{i}^{t}= & {} \frac{\sqrt{n_{i}-2 d_{i}}\left( \sqrt{d_{i}}+\sqrt{1-n_{i}+d_{i}}\right) }{\sqrt{\left( 1-\frac{n_{i}}{2}\right) n_{i}}}, \nonumber \\ g_{i j}^{s}= & {} g_{i}^{s} g_{j}^{s}, \nonumber \\ g_{i}^{s}= & {} \frac{n_{i}-2 d_{i}}{\left( 1-\frac{n_{i}}{2}\right) n_{i}}. \end{aligned}$$

In terms of these renormalization factors, the renormalized Hamiltonian can be obtained as

7 $$\begin{aligned} H^\prime =-\sum _{i\sigma }\left( g^t_{ii+x}t_x c_{i+x\sigma }^\dagger c_{i\sigma }+g^t_{ii+y}t_y c_{i+y\sigma }^\dagger c_{i\sigma }+\text {H.c.}\right) +\sum _{\langle ij\rangle } g^s_{ij}\left( \frac{J_x}{2}S_i^+ S_j^- + \frac{J_x}{2}S_i^- S_j^+ + J_zS_i^z S_j^z\right) +U\sum _{i}n_{i\uparrow }n_{i\downarrow }. \end{aligned}$$

Thus the ground-state energy in the state $\left| \Psi _{G S}\right\rangle$ can be evaluated by the expectation of $H^\prime$ in the BCS state $|\Psi _0\rangle$

8 $$\begin{aligned} E=\left\langle H^{\prime }\right\rangle _{0}=U \sum _{i} d_{i}+g_{t}\left\langle H_{t}\right\rangle _{0}+g_{s}\left\langle H_{J}\right\rangle _{0}. \end{aligned}$$

Local electron density and local pairing field, and local bond field are defined as

9 $$\begin{aligned} n_{i}= & {} \left\langle \Psi _{0}\left| c_{i \uparrow }^{\dagger } c_{i \uparrow }+c_{i \downarrow }^{\dagger } c_{i \downarrow }\right| \Psi _{0}\right\rangle =1-\delta _{i}, \nonumber \\ \Delta _{i j \sigma }^{\nu }= & {} \sigma \left\langle \Psi _{0}\left| c_{i \sigma } c_{j \bar{\sigma }}\right| \Psi _{0}\right\rangle , \nonumber \\ \chi _{i j \sigma }^{\nu }= & {} \left\langle \Psi _{0}\left| c_{i \sigma }^{\dagger } c_{j \sigma }\right| \Psi _{0}\right\rangle . \end{aligned}$$

The *d*-wave and *s*-wave superconducting gap can be given by the pairing fields and renormalization factors as

10 $$\begin{aligned} \Delta ^d_{i}= & {} \frac{1}{8} \sum _{\sigma }\left( g_{i, i+\hat{x}}^{t} \Delta _{i, i+\hat{x}, \sigma }^{v}+g_{i, i-\hat{x}}^{t} \Delta _{i, i-\hat{x}, \sigma }^{v}\right. \left. -g_{i, i+\hat{y}}^{t} \Delta _{i, i+\hat{y}, \sigma }^{v}-g_{i, i-\hat{y}}^{t} \Delta _{i, i-\hat{y}, \sigma }^{v}\right) . \end{aligned}$$

11 $$\begin{aligned} \Delta ^s_{i}= & {} \frac{1}{8} \sum _{\sigma }\left( g_{i, i+\hat{x}}^{t} \Delta _{i, i+\hat{x}, \sigma }^{v}+g_{i, i-\hat{x}}^{t} \Delta _{i, i-\hat{x}, \sigma }^{v}\right. \left. +g_{i, i+\hat{y}}^{t} \Delta _{i, i+\hat{y}, \sigma }^{v}+g_{i, i-\hat{y}}^{t} \Delta _{i, i-\hat{y}, \sigma }^{v}\right) . \end{aligned}$$

The ground-state energy can be expressed by the parameters as

12 $$\begin{aligned} E=-\sum _{\langle i j\rangle , \sigma } g_{i j}^{t} t_{i j}\left( \chi _{i j \sigma }^{\nu }+ \text{ H.c. } \right) -\sum _{\langle i j\rangle , \sigma } \frac{3}{4} J g_{i j}^{s} \Delta _{i j \sigma }^{\nu *} \Delta _{i j \sigma }^{v} -\sum _{\langle i j\rangle , \sigma } \frac{3}{4} J g_{i j}^{s} \chi _{i j \sigma }^{\nu *} \chi _{i j \sigma }^{\nu }+U \sum _{i} d_{i} \end{aligned}$$

And a mean-field Hamiltonian can be expressed as

13 $$\begin{aligned} H_{M F}= \sum _{(i j), \sigma } \varepsilon _{i j \sigma } c_{i \sigma }^{\dagger } c_{j \sigma }+ \text{ H.c. } +\sum _{\langle i j\rangle , \sigma } \sigma D_{i j \sigma }^{*} c_{i \sigma } c_{j \bar{\sigma }}+ \text{ H.c. } -\sum _{i, \sigma } \mu _{i \sigma } n_{i \sigma } \end{aligned}$$

The coefficients are given as

14 $$\begin{aligned} \varepsilon _{i j \sigma }= & {} -\frac{3}{4} J g_{i j}^{s} \chi _{i j \sigma }^{\nu *}-g_{i j}^{t} t_{i j}, \nonumber \\ D_{i j \sigma }^{*}= & {} -\frac{3}{4} J g_{i j}^{s} \Delta _{i j \sigma }^{v *}, \nonumber \\ \mu _{i \sigma }= & {} \mu -\sum _{j}\left( \frac{\partial W}{\partial g_{i j}^{s}} \frac{\partial g_{i j}^{s}}{\partial n_{i \sigma }}+\frac{\partial W}{\partial g_{i j}^{t}} \frac{\partial g_{i j}^{t}}{\partial n_{i \sigma }}\right) . \end{aligned}$$

A Bogoliubov–de Gennes (BdG) equation can solve the above Hamiltonian. Local doublon density is determined by the minimization of the ground-state energy

15 $$\begin{aligned} \frac{\partial E}{\partial d_{i}}=U+\sum _{j}\left( \frac{\partial W}{\partial g_{i j}^{s}} \frac{\partial g_{i j}^{s}}{\partial d_{i}}+\frac{\partial W}{\partial g_{i j}^{t}} \frac{\partial g_{i j}^{t}}{\partial d_{i}}\right) =0. \end{aligned}$$

The LDOS can be computed by the eigenvalues $E_n$ and eigenvectors $(u_i^n,v_i^n)$ of the BdG equation

16 $$\begin{aligned} \rho _{i}(E)=\left( g_{i}^{t}\right) ^{2} \sum _{n}\left[ \left| u_{i}^{n}\right| ^{2} \delta \left( E-E_{n}\right) +\left| v_{i}^{n}\right| ^{2} \delta \left( E+E_{n}\right) \right] . \end{aligned}$$

All the local order parameters are solved self-consistently. The system size we take is $64\times 64$. First, we input initial values to the electron density and doublon density on each site, bond field, and pairing field on each nearest-neighbor bond. Then we solve the BdG equations and calculate all the order parameters. Iterate the above process until the relative changes between the last two order parameters is less than $10^{-4}$.

## Data Availability

The data used to support the findings of this study are included in the article.
